# Subclavian Entrapment of a Seldinger Guidewire: A Rare Complication During Central Venous Catheter Placement

**DOI:** 10.7759/cureus.101943

**Published:** 2026-01-20

**Authors:** Orazio Stefano Giovanni Filippelli, Mario Pezzi, Claudio Maglia, Carmelo Romano, Teresa Valeo, Concetta Verre, Marianna De Lellis, Mary Jo Filice, Olga Sadovskaya, Stefania Faragò

**Affiliations:** 1 Anesthesia and Critical Care, Azienda Ospedaliero Universitaria ‘Renato Dulbecco’ di Catanzaro, Catanzaro, ITA; 2 Radiology, Azienda Ospedaliero Universitaria ‘Renato Dulbecco’ di Catanzaro, Catanzaro, ITA; 3 Thoracic Surgery, Azienda Ospedaliero Universitaria ‘Renato Dulbecco’ di Catanzaro, Catanzaro, ITA

**Keywords:** central venous catheterization, guidewire entrapment, procedural complication, right subclavian vein, seldinger technique

## Abstract

Central venous catheter (CVC) placement using the Seldinger technique represents a routine procedure in intensive care units; however, it is still burdened by a non-negligible risk of mechanical complications, often underestimated in clinical practice. Guidewire malposition is a rare event, but it may lead to unpredictable consequences when the course occurs in extravascular locations adjacent to osteomuscular structures. We report the case of a patient with severe cerebral hemorrhage in whom, during CVC insertion, the formation of a guidewire loop in the subclavian region occurred. The loop was completely extravascular and became entrapped in the clavicle-first rib space, making removal impossible during the procedure. This event provides an opportunity to discuss the anatomical mechanisms of malposition, the importance of early recognition, and the multidisciplinary management required to prevent iatrogenic injury.

## Introduction

Central venous catheter (CVC) placement using the Seldinger technique represents a fundamental component of intensive care practice and is widely employed for hemodynamic monitoring, administration of vasoactive drugs, parenteral nutrition, and organ support therapies. Although it is considered a routine procedure, CVC insertion is still associated with a non-negligible risk of complications, which may significantly affect patient morbidity.

Among the different access sites, the subclavian vein is often preferred because it is associated with a lower incidence of catheter-related bloodstream infections compared with the internal jugular and femoral approaches, albeit at the cost of a higher risk of mechanical complications, particularly pneumothorax [1,2]. This balance between infectious benefit and increased mechanical risk is well documented in randomized studies and international guidelines, and it requires an individualized choice of the insertion site based on patient characteristics, operator experience, and clinical context.

Mechanical complications associated with CVC placement include pneumothorax, hemothorax, arterial puncture, hematoma, catheter malposition, and cardiac arrhythmias due to excessive advancement of the guidewire or the catheter [3]. The introduction of ultrasound guidance has significantly improved procedural safety, especially for internal jugular vein access; however, the subclavian approach remains technically more challenging from an ultrasonographic perspective and is still frequently performed using anatomical landmarks, thereby maintaining a residual risk of mechanical complications.

A distinct category is represented by complications related to the guidewire itself. These include kinking, formation of loops or knots, fracture, intravascular retention, and difficulty or impossibility of removal. Although rare, such events often require advanced imaging techniques, particularly CT, to define the exact anatomical course of the guidewire and to guide safe management, and some cases may necessitate interventional radiology or surgical procedures for extraction.

In most cases described in the literature, guidewire complications are intravascular in nature. In contrast, extravascular deviation of the guidewire with loop formation and subsequent inability to remove it represents an exceptionally rare event. This mechanism implies that the guidewire exits the vascular lumen and becomes trapped within the surrounding anatomical structures. Such events are generally attributed to anatomical factors, improper needle angulation, loss of intraluminal position, or inappropriate advancement of the guidewire in the presence of abnormal resistance [4].

The costoclavicular region represents an anatomically confined and potentially critical area from a mechanical standpoint. It is well known that in this location, chronic compression of endovascular devices may occur, as described in the so-called “pinch-off syndrome” for long-term catheters. By analogy, the same anatomical district may favor mechanical impingement or entrapment of the guidewire under unfavorable conditions.

Considering the high frequency of CVC placement in intensive care and the clinical relevance of mechanical complications, the description of rare events such as extravascular deviation and entrapment of the guidewire retains important educational value. Early recognition of warning signs, such as abnormal resistance during advancement or withdrawal of the guidewire, and avoidance of forceful maneuvers are essential to prevent more serious injury.

In this context, we report a rare case of extravascular deviation of the guidewire with loop formation and costoclavicular entrapment during subclavian vein catheterization. This case highlights an unusual mechanical mechanism of guidewire incarceration and underscores the importance of careful procedural technique, immediate interruption of the procedure in the presence of abnormal resistance, and a structured diagnostic and therapeutic approach.

## Case presentation

A 77-year-old patient was admitted to the intensive care unit for a massive intraparenchymal cerebral hemorrhage, not amenable to neurosurgical treatment. During hospitalization, the patient required mechanical ventilation in pressure-controlled mode and high-dose vasopressor support because of severe hemodynamic instability.

Right subclavian CVC placement was performed using a landmark-guided technique. Ultrasound guidance was not employed for venous puncture or for real-time guidewire advancement. However, bedside ultrasound examination of the neck vessels was performed to exclude guidewire migration into the internal jugular vein, thus indirectly confirming the correct caudal direction of the guidewire toward the superior vena cava. The needle was inserted at the mid-clavicular third, approximately 1 cm below the inferior margin of the clavicle, with an orientation parallel to the presumed anatomical course of the subclavian vein. Aspiration of dark venous blood confirmed intravascular needle positioning.

The guidewire was introduced through the syringe plunger supplied with the CVC kit, without encountering resistance during advancement. However, when the guidewire had been advanced to approximately two-thirds of its length, no ectopic cardiac activity was observed on electrocardiographic monitoring, as would normally be expected when the guidewire reaches the right atrium. In addition, bedside ultrasound examination of the neck did not reveal the presence of the guidewire within the internal jugular vein.

Assuming correct intravascular positioning, catheter advancement over the guidewire was attempted. During this step, marked resistance was perceived beneath the clavicle. Guidewire withdrawal was therefore attempted; however, despite gentle traction, complete removal proved impossible, indicating entrapment of the distal portion of the guidewire.

Imaging studies

Chest Radiography

**Figure 1:**
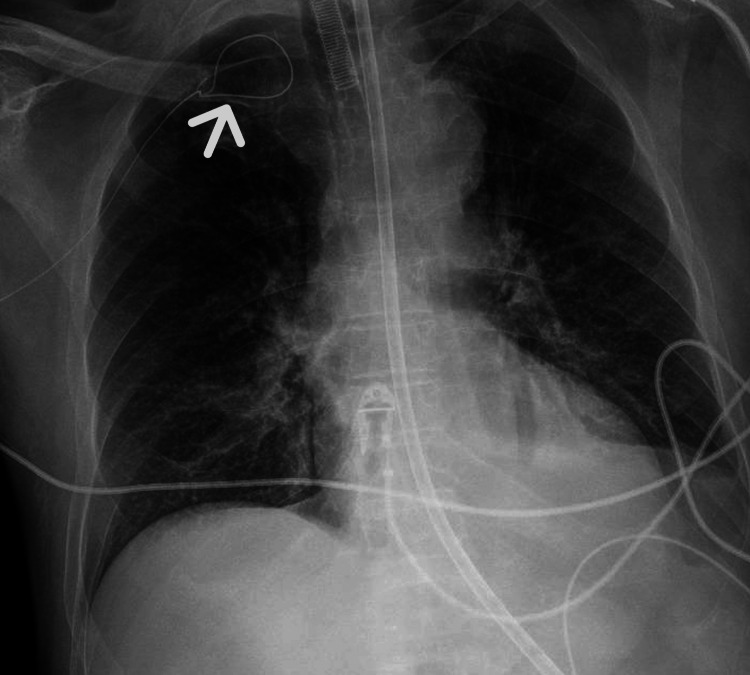
Chest X-ray Chest X-ray showing a looped configuration of the guidewire in the right subclavian region. The arrow indicates the abnormal loop formation beneath the clavicle, suggesting guidewire malposition and prompting further radiological evaluation.

Chest X-ray demonstrated a looped configuration of the guidewire in the subclavian region, prompting further radiological investigation (Figure 1).

CT

**Figure 2:**
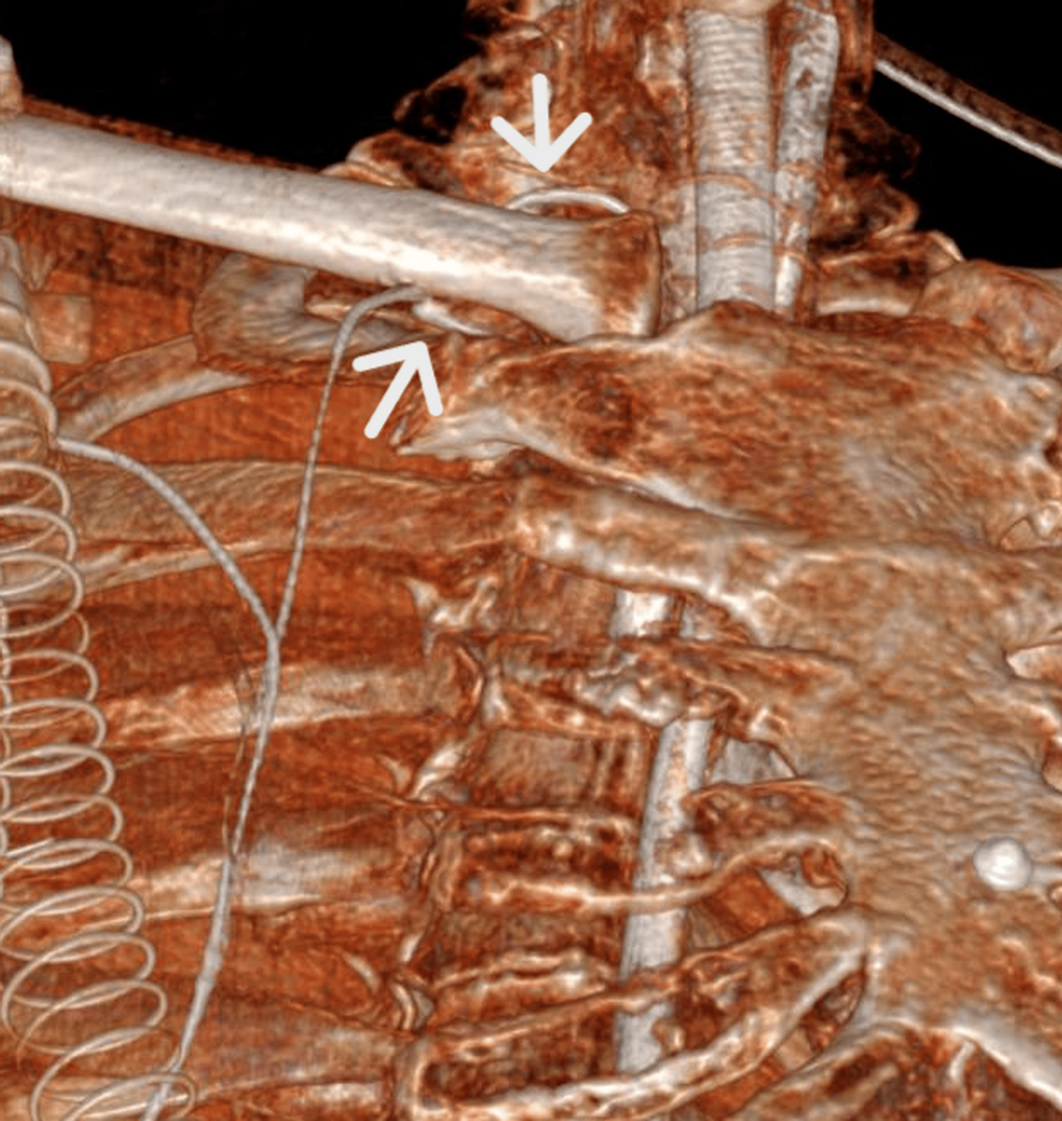
Three-dimensional CT reconstruction Three-dimensional CT reconstruction demonstrating guidewire entrapment in the costoclavicular region. The upper arrow indicates the point where the guidewire bends beneath the clavicle, while the lower arrow shows the extravascular course of the guidewire along the posterior margin of the clavicle, clearly depicting the mechanism of mechanical incarceration.

To better define the anatomical course of the guidewire, CT was performed. Three-dimensional CT reconstruction clearly demonstrated entrapment of the guidewire within the costoclavicular space, with bending and compression between the clavicle and the first rib (Figure 2).

**Figure 3:**
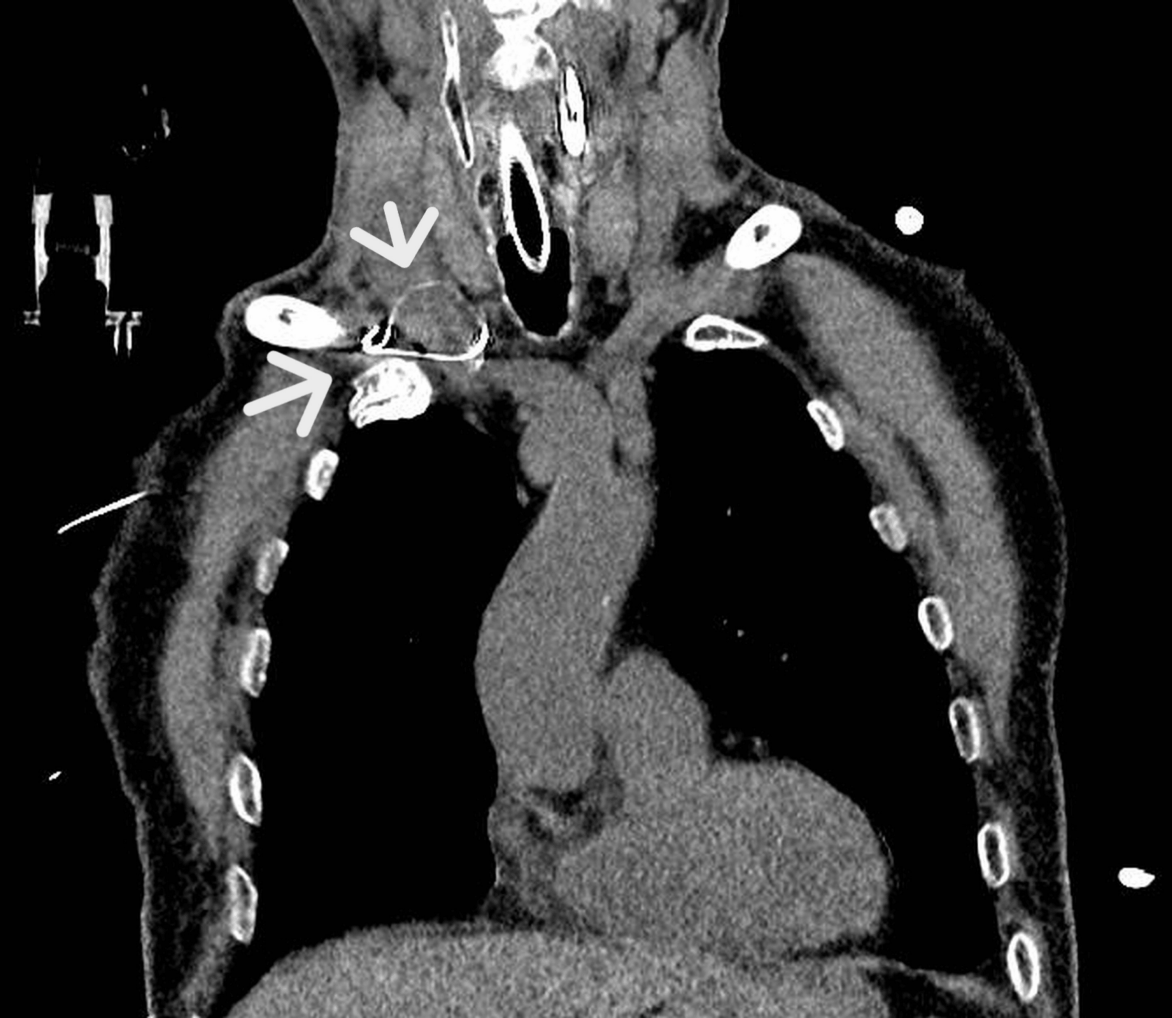
Coronal CT scan Coronal CT scan illustrating the extravascular trajectory of the guidewire. The arrows indicate the guidewire outside the vascular lumen, anterior to the subclavian vein, and its relationship with the surrounding anatomical structures, confirming extravascular deviation and costoclavicular entrapment.

Coronal CT images confirmed the extravascular trajectory of the guidewire and its relationship with the surrounding anatomical structures, supporting its localization outside the vascular lumen (Figure 3).

**Figure 4:**
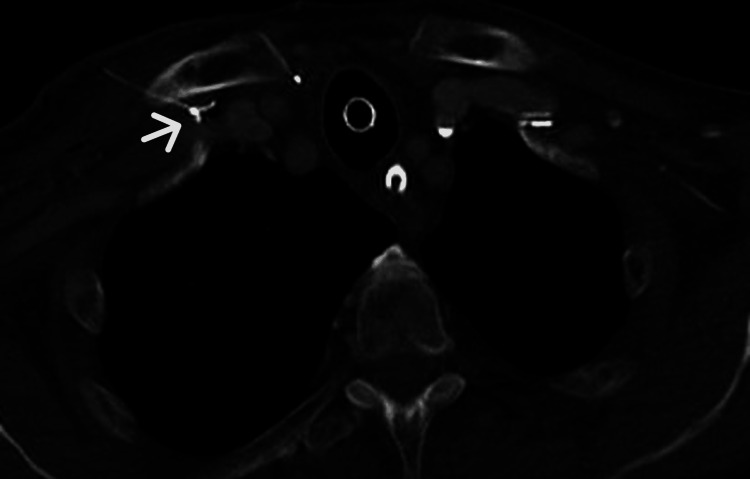
Axial contrast-enhanced CT scan Axial contrast-enhanced CT scan showing the guidewire compressed within the costoclavicular space. The arrow indicates the guidewire trapped between the clavicle and the first rib, providing direct and definitive evidence of mechanical entrapment.

Finally, an axial contrast-enhanced CT scan provided direct and definitive evidence of guidewire compression between the clavicle and the first rib within the costoclavicular space (Figure 4).

## Discussion

The present case represents one of the rarest mechanical complications associated with CVC placement. Although resistance during guidewire advancement is commonly considered an early warning sign of malposition, its absence does not exclude extravascular deviation, particularly in regions characterized by anatomical complexity and tissue laxity [4].

In this case, two subtle but relevant signs suggested guidewire malposition: the absence of ectopic cardiac activity during guidewire advancement and the abnormal resistance encountered during catheter insertion. Both findings have been described as indicators of an incorrect guidewire trajectory during CVC placement [3].

The costoclavicular region represents a particularly vulnerable anatomical area, where guidewire compression between the clavicle and the first rib may occur. Rare cases of guidewire entrapment related to costoclavicular compression have been previously reported, supporting the proposed mechanism observed in this patient [5].

This case highlights that even when the procedure is performed correctly, rare mechanical complications may still arise due to intrinsic anatomical constraints. The landmark-based infraclavicular subclavian approach, when executed by experienced operators, has historically demonstrated a low incidence of pleural complications. However, the costoclavicular space represents an anatomical bottleneck where guidewire compression or entrapment may occur independently of operator expertise. Therefore, this complication should be regarded as an anatomical risk inherent to the subclavian route rather than a failure of technique.

Predisposing factors for guidewire malposition include steep needle angulation and anterior deviation of the guidewire relative to the venous axis. Although real-time ultrasound guidance is generally recommended to reduce these risks [6], its effectiveness in the infraclavicular subclavian approach is intrinsically limited by the acoustic shadow of the clavicle. The costoclavicular segment, therefore, remains partially inaccessible to direct sonographic visualization, even in fully ultrasound-guided procedures. This anatomical constraint explains why rare mechanical complications such as guidewire entrapment may still occur despite optimal technical execution.

Once extravascular guidewire entrapment was radiologically confirmed, a multidisciplinary approach involving thoracic and vascular surgeons was promptly initiated. However, given the technical difficulty of surgical removal and the patient’s extremely poor neurological prognosis, no further invasive intervention was undertaken.

## Conclusions

Extravascular guidewire malposition with subclavian entrapment represents an exceptionally rare but potentially serious complication of CVC placement. Early recognition of abnormal procedural signs, appropriate use of ultrasound when technically feasible, and timely performance of CT play a central role in establishing an accurate diagnosis and ensuring safe management of this complication. In light of the intrinsic anatomical limitations of sonographic visualization in the costoclavicular region, this event should be interpreted as an anatomical risk inherent to the subclavian approach rather than as a failure of technique. When guidewire entrapment occurs, a multidisciplinary approach is essential to minimize the risk of further iatrogenic injury.
